# Experimental Investigation of an Intensified Heat Transfer Adsorption Bed (IHTAB) Reactor Prototype

**DOI:** 10.3390/ma14133520

**Published:** 2021-06-24

**Authors:** Karolina Grabowska, Anna Zylka, Anna Kulakowska, Dorian Skrobek, Jaroslaw Krzywanski, Marcin Sosnowski, Katarzyna Ciesielska, Wojciech Nowak

**Affiliations:** 1 Faculty of Science and Technology, Jan Dlugosz University in Czestochowa, Armii Krajowej 13/15, 42-200 Czestochowa, Poland; a.zylka@ujd.edu.pl (A.Z.); a.kulakowska@ujd.edu.pl (A.K.); d.skrobek@ujd.edu.pl (D.S.); j.krzywanski@ujd.edu.pl (J.K.); m.sosnowski@ujd.edu.pl (M.S.); k.ciesielska@ujd.edu.pl (K.C.); 2 Faculty of Energy and Fuels, AGH University of Science and Technology, Mickiewicza 30 St., 30-059 Krakow, Poland; wnowak@agh.edu.pl

**Keywords:** silica gel, porous media, adsorption bed, fluidized bed, low-pressure sorption, heat transfer

## Abstract

The first experience in the operation of intensified heat transfer adsorption bed reactor designed for low-pressure adsorption processes is presented in this paper. This work aims to assess the possibility of fluidizing the porous media bed induced by the pressure difference between the evaporator and the adsorption reactor. The conducted experimental research allowed indicating the type of silica gel recommended to use in fluidized beds of adsorption chiller. The fixed bed of silica gel was observed for the lower pressure differences, while fluidization appeared in the case of the pressure difference between the evaporator and the adsorption chamber higher than 1000 Pa. The most significant differences in the adsorption process between the fixed bed and the fluidized bed are revealed in the changes of sorbent temperatures. The silica gel bed was fluidized with water vapor generated in the evaporator.

## 1. Introduction

**Figure 1:**
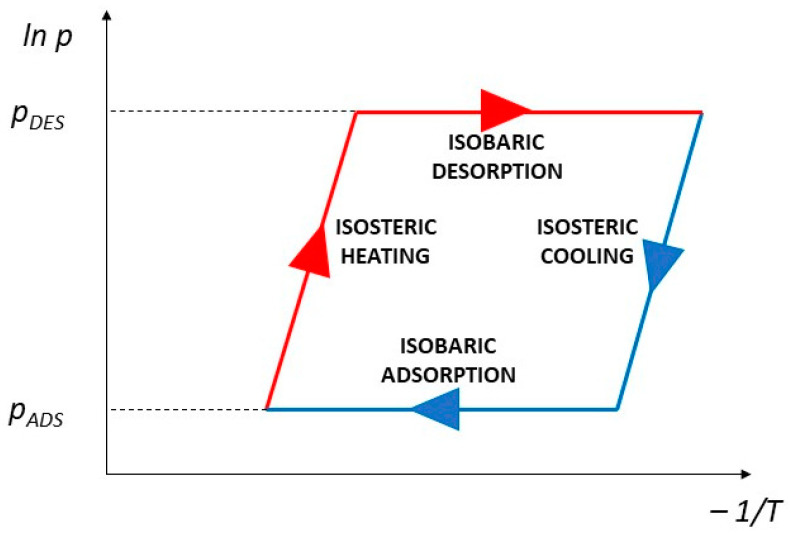
Diagram of pressure and temperature changes in the sorption cycle of the adsorption chiller’s bed.

The optimal conditions of the proecological endeavors have been discussed internationally for several decades [1]. According to the concluded global climate regulations, economic growth in the 21st century has to comply with the sustainable development concept [2]. The increasing electricity demand led to excessive exploitation of natural resources, justified by higher production efficiency. Currently, in the face of global warming, many actions are taken to improve the industry’s energy efficiency. One of the critical tasks in this area is the diversification of energy sources. Low-carbon technologies are currently being implemented in many industries, including heating and cooling technologies [3,4]. The adsorption and absorption chillers are included as innovative and ecological solutions for refrigeration. In adsorption units, the cooling effect is produced during periodically conducted sorption cycles in a bed of highly porous media, according to Figure 1.

It is known to use such devices to chilled water production in air conditioning systems [5,6]. There are also attempts to use them in food storage that requires low and stable temperatures and for the ice-making in the food industry [7,8]. Moreover, laboratory tests of mobile refrigeration devices are carried out [9]. To fully popularize the use of adsorptive cooling, it is necessary to develop effective methods to increase the efficiency of these devices, which is currently significantly lower compared to compressor chillers [10,11]. According to the literature, the maximum coefficient of performance (COP) of adsorption chillers usually ranges from 0.5 to 0.6, while the COP of the compressor refrigerator reaches even 5 [12]. The possibility of utilizing industrial waste heat and other low-temperature energy sources to supply adsorption devices makes it necessary to conduct optimization studies that will allow for introducing to the market the highly efficient ecological competition for conventional cooling systems powered by electricity [13,14]. In [15], using adsorption chillers for utilizing waste heat from power plants has been proposed. Additional areas of application of the adsorption technology to water desalination or long-term thermal storage increase its attractiveness regarding the assumptions of the low-emission economy that the world is aiming for. Many different optimization methods are considered to improve the heat transfer conditions in adsorption devices. The possibility of using new adsorptive materials to fill the bed is widely discussed in the literature [16]. In [17,18], the potential of metal-organic frameworks due to their highly developed specific surface area up to 6000 m^2^/g was presented. Other porous structures like calcium chloride, silico-alumino-phosphates SAPO and advanced composite adsorbents were analyzed in [19,20] especially in terms of their low regeneration temperature (below 100 °C), higher thermal conductivity, favorable shaped isotherm, mechanical and hydrothermal stabilities. The measurements of properties and performance prediction of the new MWCNT-embedded (multiwalls carbon nanotubes) zeolite have been conducted in [21]. Many experimental tests have analyzed the coated layer and material additives on the sorption capacity and heat and mass transfer conditions. The process of developing innovative coated bed construction was shown in [12,22]. The adhesive additives on silica gel adsorption beds were investigated in [23]. Due to the possibility of increasing the total thermal conductivity of the bed, the beneficial effect of metals and carbon nanotubes has been analyzed in [24,25] as the mixtures with the silica gel as the base sorbent.

The intensive development of advanced computational methods creates new possibilities of optimization both the bed’s design and the operating cycle conditions of the adsorption device. The numerical analyses of the geometry variation influence of porous media on the adsorption process were studied in [26]. The studies were conducted on water-silica gel pair and the adsorbent’s particle size in a fixed bed was the considered parameter. The artificial intelligence (AI) algorithms were implemented in [27,28] to optimize adsorption systems operation. The AI methods can also be successfully used to model multibed chillers with multistage working cycles, in which the interdependence of many parameters determines the total efficiency of the device and the generated cooling capacity [29]. The computational fluid dynamics (CFD) models were elaborated in [30,31] to create and evaluate the novel constructions of adsorption beds. The numerical analyses of the finned heat exchanger geometries were carried out in [32,33] in order to improve the heat transfer. However, the potential of numerical computation can also be used to design and predict the performance of novel adsorption systems with fluidized beds that have not yet been implemented on an industrial scale in the refrigeration sector [34].

Fluidization is the state of creating a dynamic suspension, so-called fluidized bed, which constitutes fine solid particles in a stream of gas or liquid has long been utilized in high-pressured boilers and reactors in order to intensify the chemical and physical processes [35,36]. Fluidized technology is used in large-scale industrial boilers, where utilizing circulating fluidized bed combustors improves combustion efficiency [37,38]. Moreover, the use of a chemical looping combustion technology based on fluidization allows for the efficient combustion of fossil fuels while achieving lower air pollution emission [39,40]. In paper [41], a dehumidification system with a circulating fluidized bed was designed to achieve high dehumidification performance and continuous operation by circulating desiccant particles without a motor. Researchers have already made the first attempts to describe the hydrodynamic behavior of fluidized beds operated under reduced pressure [42,43]. The first literature reports indicate the possibility of a significant improvement of heat transport conditions due to implementing of a fluidized layer in the adsorption bed [44,45].

The authors of this paper experimentally analyzed the possibility of implementing the fluidization process in adsorption chillers, which constitutes the key novelty of the carried out research. The silica gel was utilized as a porous media bed, because it is widely used in adsorption chillers in which water is the refrigerant. Hygroscopic structure, strongly developed specific surface area, adsorption/desorption in the required temperature regime enabling the use of low-temperature thermal energy sources and large participation of micropores in the porous structure of silica gel guarantee compatibility with water as an adsorbate. Moreover, being an environmentally friendly refrigerant, water provides a desalination functionality of the considered prototype [46,47]. The spherical and irregular type of silica gel was examined in fluidized bed conditions. So far, the efficiency of fluidization in adsorption systems under atmospheric pressure has been analyzed, in which the humid air with different parameters was fluidizing medium [48,49]. However, these conditions do not reflect an adsorption chiller’s real working cycle conditions based on the water–silica gel pair. Therefore, the authors took up the challenge of designing an innovative experimental stand that enables the verification of a fluidized bed in the real conditions of the adsorption chiller operating cycle, which is the essential novelty of the research. During the experimental research presented in the article, the fluidizing medium was water vapor under low pressure and not humid air at atmospheric pressure, as in the research published so far in the literature. Therefore, this article is a report on the first experiences in conducting fluidized tests using the original intensified heat transfer adsorption bed (IHTAB). This is a significant scientific step towards designing a full-size adsorption chiller with fluidized beds.

## 2. Materials and Methods

### 2.1. Test Stand

**Figure 2:**
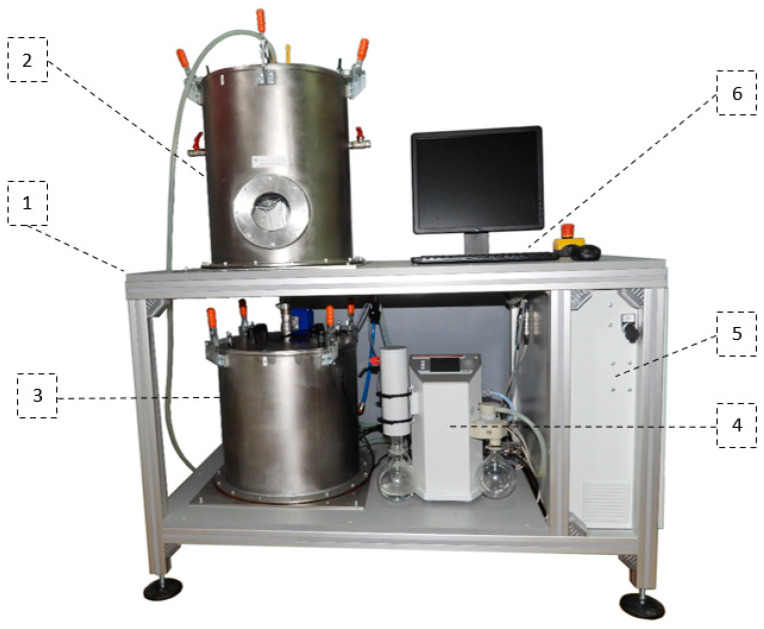
The intensified heat transfer adsorption bed (IHTAB) facility: 1—aluminum frame, 2—reaction chamber, 3—evaporator chamber, 4—vacuum pump, 5—data acquisition and control system, 6—computer.

The IHTAB prototype reactor consists of aluminum frame (1), reaction chamber (2), evaporator chamber (3), vacuum pump (4), data acquisition and control system (5) and computer (6) (Figure 2). The construction is made of Bosch Rexroth aluminum profiles. The construction enables the height adjustment of the stand ± 50 mm. The evaporator chamber is located on the lower part of the test unit. It is a sealed chamber made of stainless steel. Access to the chamber is possible by opening the hatch located in its upper part. The holding frame for temperature sensors, tensometric sensors of mass and a water tank with a heater are located inside the evaporator chamber (3).

The reaction chamber (2) is located on the top of the structure, above the evaporator chamber. It is a sealed chamber made of stainless steel, too. Access to the chamber is possible by opening the hatch located in its upper part. Outside, there are two manual water valves on the two sides of the tank and a manual shut-off valve in the upper flap. The evaporator chamber and the reaction chamber are connected with a vapor duct equipped with a separating valve.

**Figure 3:**
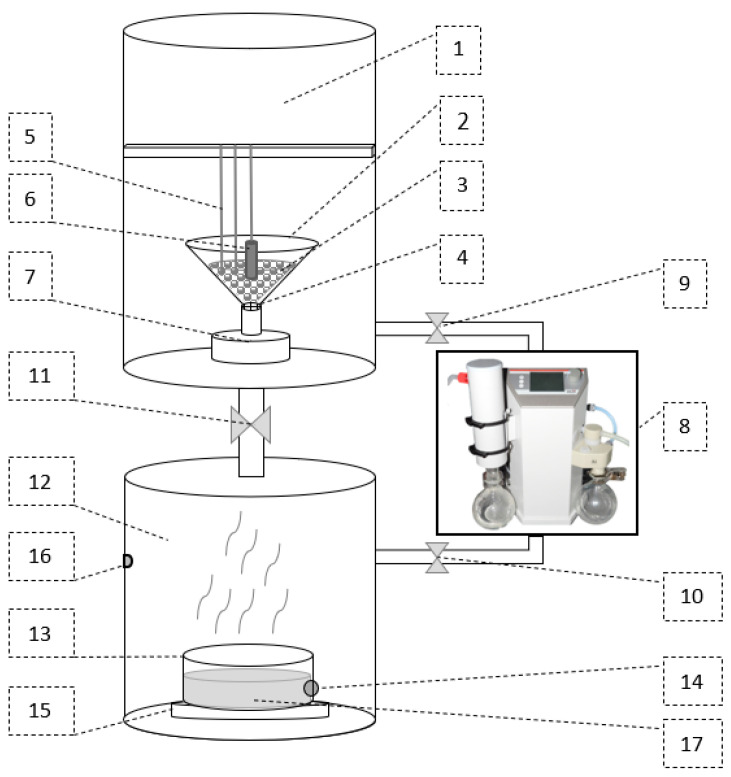
The scheme of the intensified heat transfer adsorption bed reactor: 1—reaction chamber, 2—conical spouted bed, 3—research material, 4—grid, 5—thermocouple, 6—heating probe, 7—weight measurement, 8—vacuum pump, 9—11 valves, 12—evaporator, 13—water tank with a heater, 14—temperature sensor, 15—weight measurement, 16—pressure sensor, 17—heater.

Laboratory vacuum pump (4) is placed on the lower table, next to the evaporator chamber. The pump is responsible for maintaining the conditions consistent with the working cycle of the adsorption chiller based on the water–silica gel system. The data acquisition and control system (5) has been located on the side of the unit. The test stand’s main switch, the safety reset button and the reaction chamber lighting switch are located on the door. At the front of the data acquisition and control system, there is an Ethernet and USB socket. The operator’s station (computer) (6) is located on the top table, on the right side of the reaction chamber. The detailed scheme of the IHTAB is shown in Figure 3.

The facility’s two main chambers shown in Figure 3 are evaporator (12) and reaction chamber (1). The evaporator and reaction chamber is connected to a vacuum pump (8) through the control valves (9) and (10), respectively. The connection between the evaporator and the reaction chamber is regulated by a valve (11). The amount of water in the evaporator is monitored by a tensometric weight measurement system (15). The water temperature is monitored by a temperature sensor (14) and the absolute pressure is controlled by pressure sensors (16). The evaporator is equipped with a heater (17) that allows heating the water to produce water vapor under low-pressure conditions.

The labscale model of the fluidized adsorption bed is placed in the conical spouted bed (2), while variations in the mass of this bed during the adsorption and desorption stages are monitored by the tensometric weight measurement system (7). A spouted-conical bed is one of the nonconventional fluidized beds proposed by Wen-Ching Yang in [35] to improve the parameters of beds composed of materials difficult to fluidize, e.g., due to the shape or size of the grains. Since for research hard-to-handle solids under low pressures were used, e.g., silica gel that changes its properties during vapor adsorption, carbon nanotubes that tend to agglomerate and the spouted-conical bed was used [50]. The pressure in the reaction chamber and evaporator is controlled by valves (9) and (10). The vapor saturation pressure in the evaporator and reaction chamber is determined in relation to the water temperature (14). During the measurements, the pressure in the reaction chamber is lower than the pressure in the evaporator to force the water vapor flow when valve (11) is open.

Water vapor from the evaporator flows into the reaction chamber through the adsorption bed. The mass of bed (3) change due to water vapor adsorption are recorded. The mass and pressure and temperature variations can also be observed in real-time on the user interface programmed for the needs of this original test stand.

**Figure 4:**
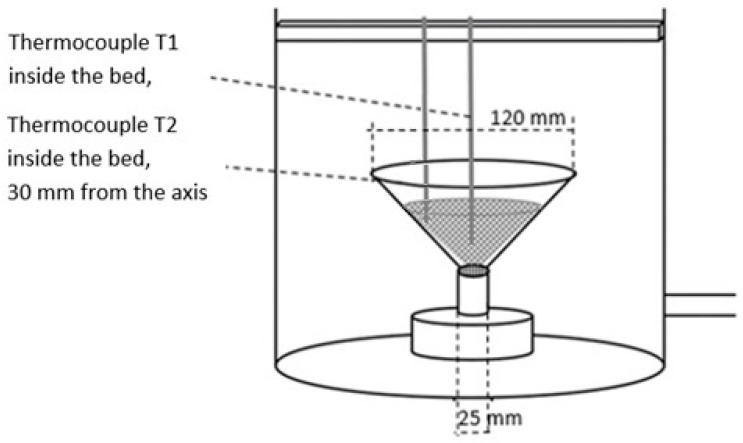
The equipment of the adsorption chamber.

This is the first study to assess the possibility of obtaining a fluidized bed state due to the pressure difference between the evaporator and the chamber. The equipment of the reaction chamber is shown in Figure 4.

### 2.2. Test Conditions and Materials

**Figure 5:**
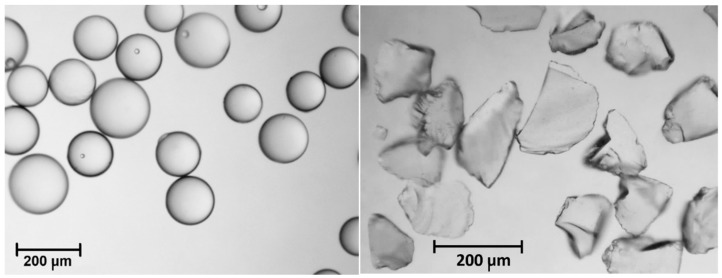
Microscopic view of the silica gels: on the left side—SG from Fuji Silysia Chemical Ltd.; on the right side—SG from Sigma Aldrich.

Two types of silica gel (SG) were selected to conduct the experimental tests as a porous material. Commercial silica gel from Fuji Silysia Chemical Ltd. (Greenville, SC, USA) and from Sigma Aldrich was used for the research. Using an Analysette 3 Spartan shaker (FRITSCH GmbH, Idar-Oberstein, Germany), the sorbent was segregated to granulation of 160–200 µm. The weight of the silica gel used in the tests was 55 g. Microscopic views of these materials are shown in Figure 5. The spherical silica gel particles from Fuji Silysia Chemical Ltd. had the following properties: the sphericity of grain 0.95, density of 2200 kg/m^3^ and bulk density of 780 kg/m^3^. Thus, the bed voidage was estimated at 0.65. The silica gel particles from Sigma Aldrich had an irregular shape with the sphericity of grain 0.65, the density of 2200 kg/m^3^ and the bulk density of 850 kg/m^3^.

During these investigations, the water vapor was used to fluidize the silica gel bed in the reaction chamber. Water vapor was generated in the evaporator. Opening valve 11 allows water vapor to migrate into the reaction chamber rapidly. The water in the evaporator was not heated during this test. Water evaporation was achieved by reducing the pressure in the evaporator with the use of a laboratory pump. An absolute pressure of about 2000–2100 Pa (20–21 mbar) was noticed inside the evaporator and the temperature of water in the tank was 21 °C. In these conditions, the water starts to boil, allowing for water vapor production, which is managed to the adsorption chamber during the sorption cycle.

The process of pressure equalization between the evaporator and the reaction chamber took approximately 10 s, and during this time, fluidization of the porous material was observed. Therefore, such a valve opening time was used for all cycles. After that, the valve was closed, and the pressure in the reaction chamber and the evaporator chamber were reduced using the laboratory vacuum pump. It took about 150 s for the pressures to return to their initial state. After this time, the cycle starts again by opening the valve for 10 s.

## 3. Results

The results from the first experimental tests are presented below. The experimental tests were carried out for two cases. The first one presented the fixed adsorption bed and is called a fixed state and the second case corresponds to the fluidized bed and is called a fluidized state.

**Figure 6:**
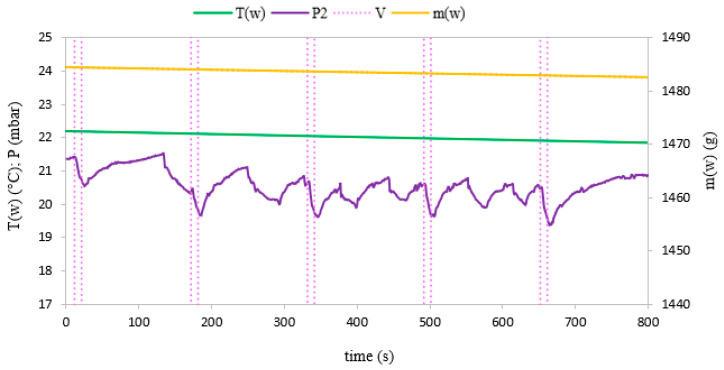
The changes of pressure, temperature and mass of evaporated water with time profiles in the evaporator corresponding to fixed bed conditions.

**Figure 7:**
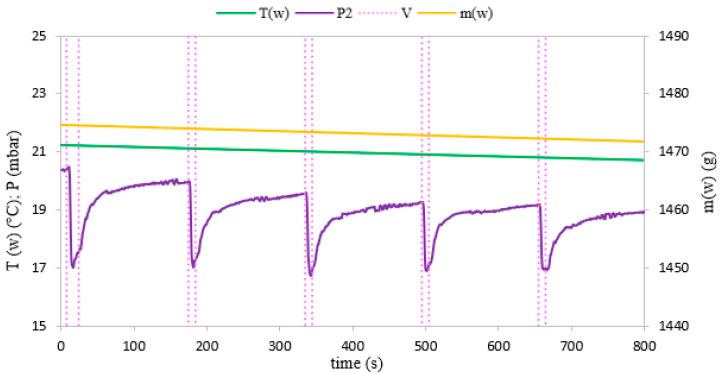
The changes of pressure, temperature and mass of evaporated water with time profiles in the evaporator corresponding to fluidized bed conditions.

Evaporation of water in the evaporator can be observed after the mass m_w_ and temperature T_w_ measurements of the water in the evaporator. These parameters change with the variation of pressure during intensive evaporation. During the 800-s test, the mass and temperature of the water decreased due to evaporation. The pressure, water mass and temperature profiles behavior for fixed and fluidized bed conditions are shown in Figure 6 and Figure 7, respectively.

**Figure 8:**
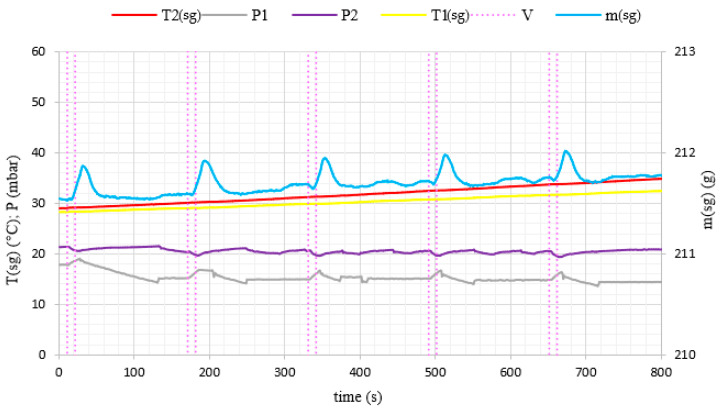
The changes of pressure, temperature and mass of evaporated water with time profiles in the adsorption chamber corresponding to fixed bed conditions (Spherical SG from Fuji Silysia Chemical Ltd.).

**Figure 9:**
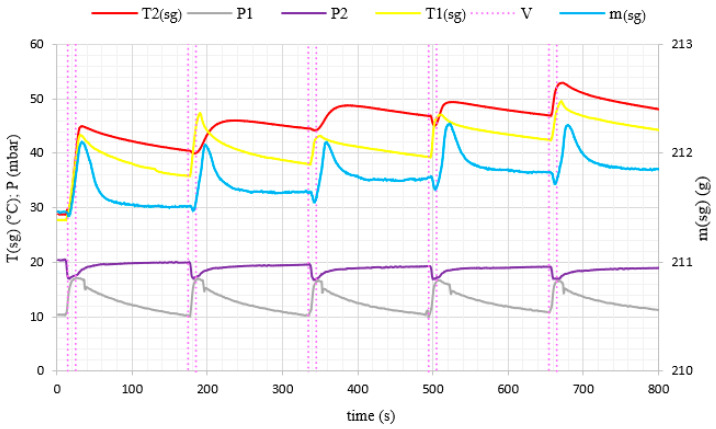
The changes of pressure, temperature and mass of evaporated water with time profiles in the adsorption chamber corresponding to fluidized bed conditions (Spherical SG from Fuji Silysia Chemical Ltd.).

**Figure 10:**
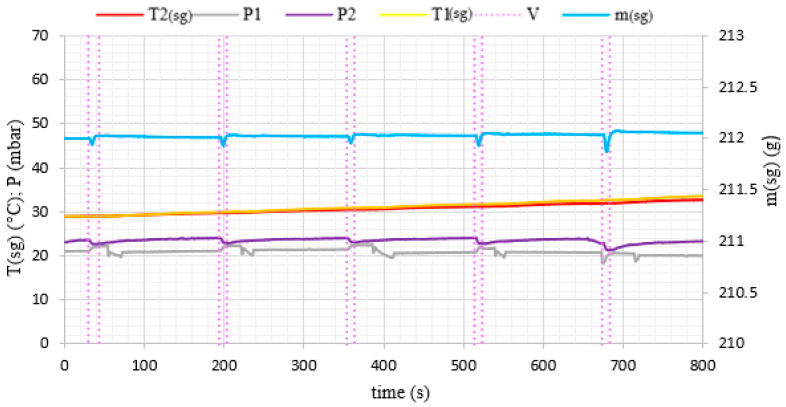
The changes of pressure, temperature and mass of evaporated water with time profiles in the adsorption chamber corresponding to fixed bed conditions (Irregular SG from Sigma Aldrich).

**Figure 11:**
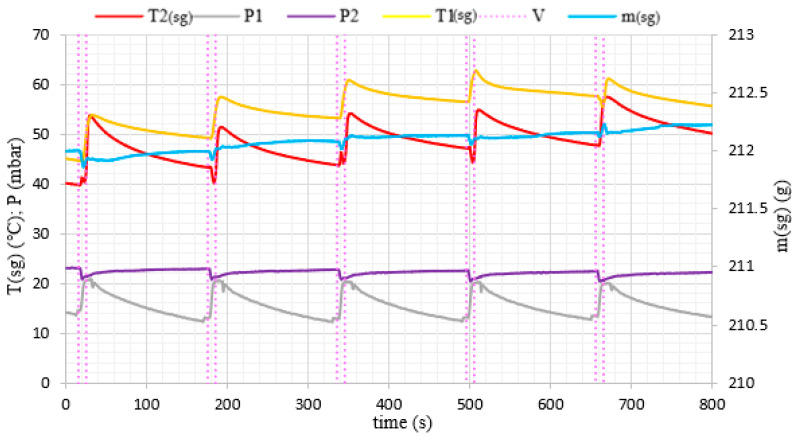
The changes of pressure, temperature and mass of evaporated water with time profiles in the adsorption chamber corresponding to fluidized bed conditions (Irregular SG from Sigma Aldrich).

During the experiments, water vapor was produced at low pressure conditions in the evaporator volume. The generated water vapor was subsequently directed to the adsorption chamber and adsorbed on the porous media. After the end of the adsorption cycle, desorption was carried out in the chamber and the desorbed vapor did not supply the evaporator again, but was removed from the system by the vacuum pump. Therefore, in Figure 6 and Figure 7, we can see a constant loss of mass of water over time because the losses in the evaporator were not replenished during the experiment. A pressure difference was created between the adsorption chamber and the evaporator. It allowed achieving a fluidized bed of silica gel in the adsorption chamber. The adsorption conditions in the fixed and fluidized states were analyzed and the influence of the sphericity of silica gel on the possibility of applying fluidization was also investigated. Figure 8 and Figure 9 show the results for Fuji Silysia silica gel of sphericity 0.95 and Figure 10 and Figure 11 show the results for silica gel from Sigma Aldrich of sphericity 0.65.

The first measurement series were conducted for the pressure difference between the evaporator and the chamber equal to 500 Pa. Fluidization was not observed at this pressure difference. The sorbent samples took the form of a fixed bed for each utilizing types of silica gel that corresponds to a conventional adsorption chiller. Measurements and profiles from this state are shown in Figure 8 and Figure 10. The fixed adsorption phenomenon was observed through the visor in the reaction chamber. The second measurements series were conducted for the pressure difference between the evaporator and the chamber equal to 1000 Pa. As shown in Figure 9 and Figure 11, the adsorption processes were more intense in these cases. Fluidization was observed at this pressure difference. The gas flow requires a pressure gradient (ΔP) between two chambers and it is directly proportional to the pressure differential. Higher pressure differences will drive higher flow rates. The pressure gradient establishes the direction of flow. According to correlations in [35,50,51], the minimum spouting velocity was equal to 0.03 m/s. Based on the comparison with reports from [35,51] the following characteristic properties of the particle movement can be defined for the conical spouted fluidized bed. At any conical spouted bed height, the particle flowing up in the spout has to be equilibrated by the particles moving down in the annulus. In the area of the fountain core, the flowing particles decelerate to reach zero velocity at the top of the fountain and fall around the ambient area. During the falling of particles, their vertical velocities increase with decreasing height due to the reduction of the conical spouted bed’s cross-sectional region.

Since valve 11 opens and fluidization in the volume of the conical spouted bed is observed, the pressures in the evaporator (P2) and reaction chamber (P1) tend to equalize. Then the mass of the silica gel bed increases due to the adsorption and the adsorption intensifies during the fluidization conditions. The mass of the bed increases because the silica gel absorbs the water vapor. Hence, it can be concluded that fluidization intensifies the processes of water vapor adsorption. After closing the valve for 150 s, the pressure in the reaction chamber is reduced. Hence the weight of the bed decreases due to the desorption process. After regeneration time, the valve is opened for 10 s and the cycle repeats.

In the case of the fluidized beds, the influence of the silica gel grains sphericity is also clearly visible. According to [35], particles of sphericity close to 1, fluidize much more easily than those of an irregular shape. It was confirmed by the rapid changes in mass profile during the opening of the Valve (11) shown in Figure 9, which presents a fluidized state for the spherical SG from Fuji Silysia Chemical Ltd. In the case of the analyzed irregular shape silica gel of sphericity equal to 0.65, presented in Figure 11, the changes in mass during the adsorption process are small, which confirms that lower sphericity hinders the fluidization process of the material [52,53].

The most significant differences in the adsorption process in the fixed and fluidized states are observed in the changes in adsorbent temperatures. The thermocouples T1_sg_ and T2_sg_, registering silica gel temperatures during adsorption, have been located in the volume of sorbent, according to Figure 4. In the cases of a fixed bed, the temperature increase is small and its character is linear throughout the entire test cycle. However, in fluidized bed conditions presented in Figure 9 and Figure 11, the temperature increase in each adsorption cycle is rapid and can even reach 22 °C compared with the initial condition, whereas the total temperature rise in the fixed bed was only 3 °C. The sharp profile of temperature changes during adsorption in the fluidized bed was observed for each analyzed type of silica gel. This relation confirms the potential of fluidization to intensify the heat transfer processes. The increase in sorbent temperature during the diffusion of water vapor is due to the exothermic phenomenon of adsorption. The analysis of the Figure 8, Figure 9, Figure 10 and Figure 11 proved that the sorption processes were more intense in the case of the fluidized bed.

## 4. Conclusions

The experimental results from the first experience of the IHTAB (intensified heat transfer adsorption bed) facility were presented in this paper. The paper presented the first study to assess the possibility of obtaining a fluidized state of porous media bed under low-pressure conditions.

The experiments required developing an innovative research stand, which constitutes a laboratory model of an adsorption chiller. It has been proven that fluidization under low-pressure conditions occurs provided that an appropriate pressure difference is achieved between the evaporator and the reaction chamber. The fixed or fluidized state in the bed of adsorption material was induced by changes in pressure differences between the adsorption chamber and the evaporator. Moreover, the presence of the fluidized state was possible because of the appropriate diameter of the porous material selection and the height of the bed. The use of a spouted-conical bed dedicated to materials difficult to be fluidized is also an essential factor. In the analyzed cases, the fluidization conditions were selected in accordance with correlations in [35,50]. Taking into account the material parameters of the silica gel and the working conditions of the IHTAB, the minimum spouting velocity was equal to 0.03 m/s. Based on literature data and experience gained during preliminary tests, the optimal pressure difference was defined to achieve a fluidized state in the silica gel bed. Moreover, the greater tendency to fluidize materials with sphericity close to 1 has been experimentally proven.

The observed periodic changes in the bed mass of spherical silica gel are higher and more dynamic, resulting from the material mixing during fluidization. In the case of the analyzed irregular shape silica gel of sphericity equal to 0.65, the changes in mass during the adsorption process are small, which confirms that lower sphericity hinders the fluidization process of the material.

The fluidized state of the adsorption bed was achieved at a pressure difference of 1000 Pa for a silica gel bed with granulation in the range of 160–200 μm. These innovative studies were carried out on a laboratory scale. Hence the observed differences of measured parameters are minor. However, the obtained results and the observed adsorption processes in the stationary and fluidized states constitute valuable information for further research scenarios. Moreover, the analysis of investigated cases proved that fluidization is a favorable phenomenon as it intensifies the processes of water vapor adsorption on silica gel porous structures.

## Data Availability

Not applicable.

## Nomenclature

P	pressure, Pa
M	mass, g
T	temperature, °C
P1	absolute pressure in the reaction chamber, mbar
P2	absolute pressure in the evaporator, mbar
V	valve
**Subscripts**	
ADS	adsorption
DES	desorption
SG	silica gel
w	water
